# Can DNA Distortion Turn RAG into a Potent Transposase?

**DOI:** 10.1371/journal.pbio.0040390

**Published:** 2006-10-24

**Authors:** Liza Gross

As a general rule, DNA rearrangements spell trouble. By facilitating the movement of genetic elements to new sites in the genome, one class of transposition enzymes—the transposase/retroviral integrase superfamily—plays a major role in disease. Transposases can cause cancer by reinserting DNA into or near cancer-related genes. Retroviral integrases pave the way for HIV infection by integrating the retrovirus into the genome. But genetic rearrangements, mediated by a recombinase produced by the recombination activating genes *(RAG)*, also underlie the body’s ability to ward off infection. By recognizing specific bits of DNA called recombination signal sequences (RSS) that bookend DNA separating two gene fragments, RAG complexes can remove the intervening DNA and join the two gene fragments remaining in the immune cell receptor gene locus. This genetic reshuffling process, called V(D)J recombination, generates the phenomenal diversity of immune cell antigen receptors that can recognize virtually any pathogen that slips into the body.

In the late 1990s, researchers discovered that the RAG complex can also act like a transposase, by reinserting DNA segments into unrelated DNA targets. This suggested that RAG-mediated transposition might trigger the chromosomal translocations seen in lymphoid tumors. But since RAG-mediated transposition was found only in “cell-free” test tube experiments, not in living cells, it was thought that cells pulled out the regulatory stops to inhibit RAG transposition and protect genomic stability.

In a new study, Jennifer Posey, David Roth, and colleagues show that RAG can mediate transposition quite effectively—provided the right target is available. Their findings could explain why researchers have had such a hard time finding evidence of RAG transposition in living cells.

Because transposases often exhibit clear biases for certain DNA targets, Posey et al. suspected that target-site selectivity might provide the regulatory means to block RAG transposition without preventing its V(D)J recombination activity. Early studies suggested that RAG transposition preferentially targets stretches of DNA rich in guanine (G) and cytosine (C) nucleotides, especially certain GC hotspots. But more recent evidence indicates that RAG transposition favors distorted DNA structures called hairpins—single-stranded DNA that folds back on itself to form a loop—at the tips of a “stem” of nucleotides. (When this “stem and loop” structure forms on both strands of DNA, it is called a cruciform.)

Because the last four nucleotides of a hairpin provide targets for other DNA-cleaving enzymes (called endonucleases), the authors thought the terminal ends of hairpins might do the same for RAG transposition. To investigate this possibility, they generated a set of 16 DNA fragments, covering all possible four-nucleotide combinations around the hairpin tip, each having the same stem and a different hairpin tip. They incubated each tip with RAG proteins and RSS-bounded DNA segments and calculated transposition efficiency as the percentage of RSS ends transposed into the hairpin target.

**Figure:**
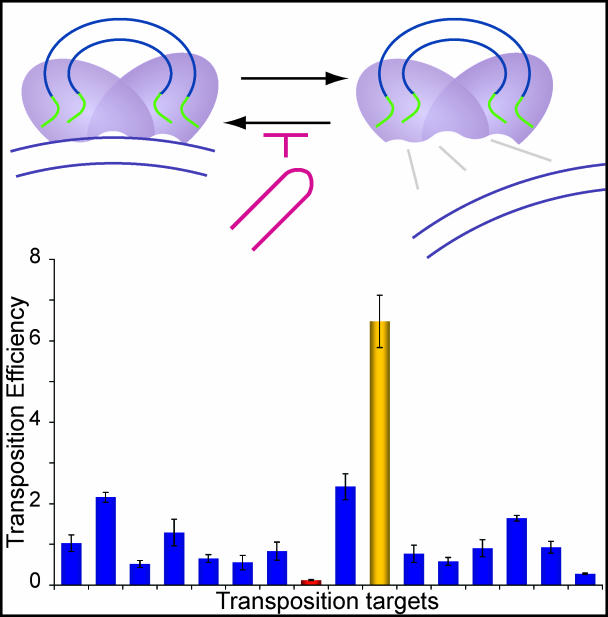
RAG transposition, thought to be rare, is actually robustly stimulated by the correct hairpin targets. One structure, however, inhibits transposition by preventing target capture.

Transposition efficiency ranged from “virtually undetectable” to “robust,” depending on the tip’s nucleotide sequence. Still, most of the hairpins acted as strong targets. Interestingly, GC tips generated far more activity than CG, indicating that transposition depends on more than nucleotide content alone. Rather, the sequence of the four nucleotides around the hairpin determines the structure of the tip and thus how attractive a target it will be for RAG transposition. When the nucleotide sequences support a cruciform structure, they stimulate the most efficient transposition.

The exception to the rule is the CT (cytosine-thymine) hairpin, which actually inhibited transposition, even though it did not inhibit the RAG proteins’ ability to cleave DNA and could bind to the RAG/RSS complex. Interestingly, a CT sequence that did not adopt a cruciform structure had no inhibitory effect on transposition. It may be that the CT hairpin interferes with RAG activity by somehow preventing the RAG complex from successfully capturing the target—a possibility that can be explored in future experiments.

By showing in the test tube that the RAG complex can readily stimulate transposition when it encounters a preferred target, this study should stimulate new searches for RAG transposition in living cells. Given the RAG proteins’ highly specific target preferences, it’s not surprising that RAG transposition has been so hard to find in living cells. But now that researchers have a clearer idea of what to look for, they can look for the telltale signs of RAG transposition in lymphoid tumors to shed light on its potential contributions to cancer.

